# Probabilistic Finite Element Modeling of Textile Reinforced SHCC Subjected to Uniaxial Tension

**DOI:** 10.3390/ma14133631

**Published:** 2021-06-29

**Authors:** Iurie Curosu, Amr Omara, Ameer Hamza Ahmed, Viktor Mechtcherine

**Affiliations:** Institute of Construction Materials, Technische Universität Dresden, 01062 Dresden, Germany; amromara.civil@gmail.com (A.O.); ameer_hamza.ahmed@tu-dresden.de (A.H.A.); viktor.mechtcherine@tu-dresden.de (V.M.)

**Keywords:** SHCC, textile reinforcement, bond, FEM, probabilistic model, multiple cracking

## Abstract

The paper presents a finite element investigation of the effect of material composition and the constituents’ interaction on the tensile behavior of strain-hardening cement-based composites (SHCC) both with and without textile reinforcement. The input material parameters for the SHCC and continuous reinforcement models, as well for their bond, were adopted from reference experimental investigations. The textile reinforcement was discretized by truss elements in the loaded direction only, with the constitutive relationships simulating a carbon and a polymer textile, respectively. For realistic simulation of macroscopic tensile response and multiple cracking patterns in hybrid fiber-reinforced composites subjected to tension, a multi-scale and probabilistic approach was adopted. SHCC was simulated using the smeared crack model, and the input constitutive law reflected the single-crack opening behavior. The probabilistic definition and spatial fluctuation of matrix strength and tensile strength of the SHCC enabled realistic multiple cracking and fracture localization within the loaded model specimens. Two-dimensional (2D) simulations enabled a detailed material assessment with reasonable computational effort and showed adequate accuracy in predicting the experimental findings in terms of macroscopic stress–strain properties, extent of multiple cracking, and average crack width. Besides material optimization, the model is suitable for assessing the strengthening performance of hybrid fiber-reinforced composites on structural elements.

## 1. Introduction

Textile reinforced concrete (TRC) and fabric reinforced mortars (FRM) are cementitious composites reinforced with continuous, two-dimensional, or three-dimensional textiles/fabrics made of carbon, alkali-resistant glass, or polymer multifilament yarns [1,2,3,4]. These composites feature controlled multiple cracking under increasing deformation prior to reaching the tensile strength of the loaded yarns and are suitable as externally applied retrofitting and strengthening layers on concrete and masonry elements [5]. The mechanical and physical properties of the reinforcing yarns allow the achievement of effective and durable strengthening properties in narrow thicknesses of up to 20 mm.

Although highly promising for applications involving ordinary service conditions, the energy dissipation capacity and damage tolerance of TRC/TRM require further enhancement for scenarios involving dynamic or repeated mechanical loading with extensive deformations and high energy input [6]. This can be achieved through the targeted addition of short fibers, which can increase the cracking stress of the cementitious matrix, decrease the crack spacing and crack width, and mitigate matrix spalling [7,8].

Another promising material for such strengthening applications exists in the form of strain-hardening cement-based composites (SHCC), also known as engineered cementitious composites—ECC. SHCC are made of fine-grained cementitious matrices and short polymer micro-fibers, and they exhibit a strain-hardening tensile behavior with pronounced multiple cracking prior to failure localization [9]. Despite their high strain capacity, high energy dissipation potential, and damage tolerance, SHCC demonstrate relatively low tensile strength, and their mechanical performance in real-scale applications can be negatively affected by production and application techniques [10] and boundary conditions and structural size as well [11,12]. Furthermore, the multiple cracking in SHCC degrades stiffness, a development that can limit the degree of confinement offered by such strengthening layers without continuous reinforcement.

In this context, the combination of SHCC with textile reinforcement can effectively mitigate the disadvantages of each composite in particular and ensure superior mechanical features both under quasi-static [13,14] and impact loads [15,16]. However, aside from the mechanical features, the sustainability factors, especially the economical and the ecological ones, are essential with regard to the real-scale applicability of such materials. An economical material design requires the purposeful selection of the constituents and adjustment of their interaction with respect to specific performance requirements. Given that the experimental investigations of such composites imply high financial costs and require advanced technical equipment, high-fidelity numerical simulations are essential in enabling a wide and detailed study of various hypotheses and material parameters. Furthermore, these should serve as a reliable basis for assessing the strengthening performance of such composites at the structural level.

In order to analyze and predict accurately the influence of composition, application, and boundary conditions on the strength, deformation, and fracture properties of fiber-reinforced composites, the material models should imply reinforcement discretization both for textile [17,18] and short fibers [19]. Finite element (FE) [19] and discrete lattice models with discrete fiber representation [20,21,22] imply the explicit consideration of the spatial inhomogeneity and anisotropy related to fiber distribution and orientation, but the substantial computational effort caused by the geometric properties of discrete fibers limits the applicability of this approach to small regions of interest [23,24].

For morphologically detailed but computationally efficient numerical simulations of hybrid fiber-reinforced composites, a continuum representation of the multiple cracking in SHCC can be adopted [25,26,27,28], while the inherent material variability can be accounted for within a probabilistic representation of the relevant material parameters, such as matrix strength and tensile strength [29]. For applications requiring the accurate modeling of localized fracture, discrete crack models can be adopted or combined with the smeared crack model [29,30].

Commonly, the probabilistic approach is applied in phenomenological and numerical models of plain SHCC [26,27], while the continuous reinforcement is discretized in the context of steel-reinforced concrete elements [25]. The paper at hand presents a FE model that combines these two approaches in the context of hybrid fiber-reinforced composites, i.e., the probabilistic approach for SHCC and the discrete representation of the continuous textile yarns. It enables a detailed analysis of the interaction mechanisms and of the effect of various material combinations on the macroscopic composite properties and facilitates an accurate prediction of the multiple cracking and fracture phenomena in the tensioned composites. The presented approach is applicable both to material design and optimization purposes as well as to assessing the structural behavior of elements strengthened with such composites.

The model and the numerical study presented in this paper complement a series of experimental investigations aimed at clarifying the influence of the constituents’ properties and their interaction on the mechanical behavior and strengthening performance of SHCC with textile reinforcement [13,14]. Given that the primary focus was laid on the pre-peak tensile properties of such composites, the numerical material model of SHCC implied a smeared crack formulation of both the strain-hardening and localization phases. The mesh-size sensitivity within the smeared crack model was counteracted by the probabilistic representation and spatial fluctuation of the material parameters equivalent to the matrix strength and collective crack-bridging strength in SHCC. The main goal of the probabilistic formulation was a realistic representation of the successive and spatially distributed multiple cracking in SHCC, depending on the inherent material variability in terms of flaw size distribution [31,32] and fiber orientation and distribution [33,34]. Moreover, the interaction with the continuous reinforcement was another important aspect analyzed with regard to the resulting extent of multiple cracking and crack width [13,14]. Following the multi-scale concept [29,35], the constitutive law of SHCC reflected the single-crack opening behavior. The adjustment of the probabilistic distribution of the matrix strength and tensile strength in SHCC allowed fitting the effective extent of multiple cracking of the modeled composite specimens.

The continuous reinforcement was discretized by truss elements embedded in the longitudinal (loading) direction of the composite specimens under tension. The influence of stiffness and elongation capacity of the continuous reinforcement, as well as of the bond strength between the continuous reinforcement and the SHCC, were analyzed in a parameter study. Besides the macroscopic tensile stress–strain relationships of the plain SHCC and of the hybrid fiber-reinforced composites, the multiple cracking pattern and the resulting crack width along the entire deformation range were analyzed and compared to the reference experimental results presented in [13,14].

## 2. Finite-Element Model

**Figure 1:**
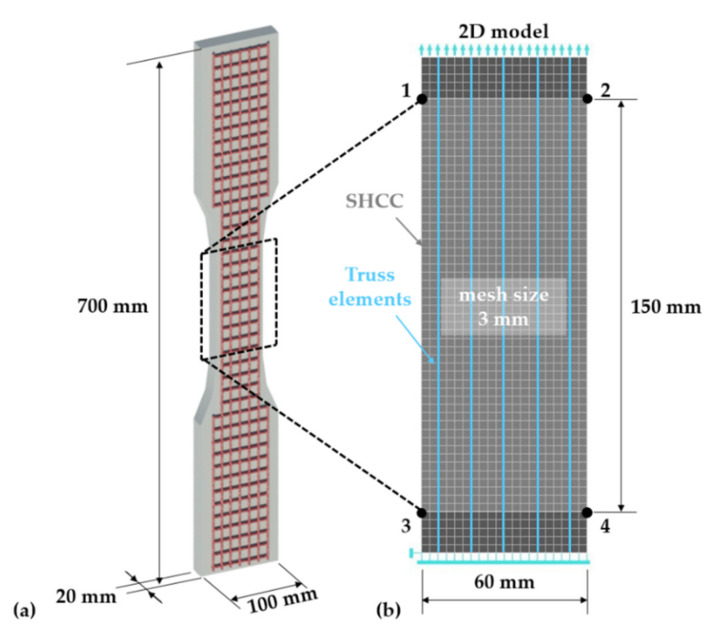
(**a**) Geometry and dimensions of the composite specimens with one layer of textile reinforcement and (**b**) mesh size and boundary conditions of the modeled region of interest in the 2D simulations.

The finite-element simulations were performed using the software Atena Engineering 2D V5.6.1, by Cervenka Consulting s.r.o (Prague, Czech Republic). The constitutive nature and geometry of the modeled composite specimens allowed performing 2D simulations to limit the computational effort. The geometry and dimensions of the modeled composite specimens are presented in Figure 1a according to [13,14]. The modeled region of interest corresponds to the gauge portion, having a width of 60 mm and length of 150 mm; see Figure 1b. The 20 mm thickness of the experimental specimens defined the nominal thickness of the solid finite elements in the 2D simulations. The mesh size of 3 mm was defined according to the minimum average crack spacing observed in tensioned composite specimens with hybrid fiber reinforcement [13,14]. It was assumed that this crack spacing corresponds to the crack saturation achievable by the material compositions analyzed with the given specimen geometry and boundary conditions. The same applies to the defined stress parameters of the SHCC model.

The hybrid fiber-reinforced specimens accommodated five longitudinal yarns as presented in Figure 1b. The truss elements were arranged over the nodes of the SHCC mesh, resulting in a spacing of 12 mm, slightly smaller than the spacing of the warp yarns in the actual textile of 12.7 mm [13]. The degrees of freedom (DOFs) of the truss elements are independent of the DOFs of the underlying solid elements. Given the textile production technology of the reinforcing textiles by stitch bonding, the joints between the warp (longitudinal) and weft (transversal) yarns are neither rigid nor strong. In the context of uniaxial tensile loading, this allowed discretizing only the warp yarns. The weft yarns might contribute to the anchorage strength of the warp yarns, but this was implicitly considered by defining the bond properties based on warp–yarn pullout experiments with attached weft yarns [14]. Furthermore, the weft yarns might weaken the corresponding cross-sections of the composite specimens, but this effect cannot be captured with transversal truss elements, and it was taken into account by the probabilistic representation of the matrix strength.

In the experimental specimens, the ends of the textile yarns embedded in the anchorage portions, aside from gauge length, were coated with epoxy-resin and sand in order to limit yarn slip. However, the additional coating did not ensure a perfect bond, and the deformation measurements on the gauge portion included the yarn delamination and partial pullout from the anchoring portions [14]. Thus, disabling the relative slip of the truss elements at the ends of the gauge portion would not accurately reflect the real boundary conditions in the experiments. For this reason, additional anchorage portions with lengths of 15 mm, i.e., five elements, were defined at the ends of the gauge portion; see the darker solid elements in Figure 1b. The additional solid elements had an identical Young’s modulus to the core SHCC elements but were designed to deform elastically throughout the entire load range. The slip of the truss elements relative to the solid elements was disabled at the boundary nodes.

The model specimen was loaded by a prescribed deformation of 0.1 mm with a step multiplier of 0.03 at the nodes of the top boundary elements, and the reaction forces were recorded and summed at the nodes of the bottom row, as presented in Figure 1b. Only the deformation of the 150 mm long gauge portion was considered in deriving the macroscopic specimen deformation according to the relative displacement of the solid element nodes 1,2 and 3,4, as presented in Figure 1b. The standard Newton Raphson method was used as the solution procedure with an iteration number limit of 40.

### 2.1. SHCC—Finite-Element Formulation

SHCC was discretized with plane quadrilateral elements of type CCIsoQuad, which are is oparametric elements featuring bilinear interpolation and four Gaussian integration points [36]. The CC3DNonLinCementitious2User material model was applied to model SHCC due to the possibility of model strain-hardening. This material model is a fracture-plastic constitutive model, combining tensile (fracture) and compressive (plastic) behavior. The fracture model is based on the classical orthotropic, smeared crack formulation, and crack band model [36,37]. The hardening/softening plasticity model is based on the Menetrey–Willam failure surface. The tensile strain is decomposed in its elastic, plastic, and fracture components, as shown in Equation (1):

(1) $$\varepsilon_{ij}=\varepsilon_{ij}^{e}+\varepsilon_{ij}^{p}+\varepsilon_{ij}^{f}$$

with the new stress state computed as in Equation (2):

(2) $$\sigma_{ij}^{n}=\sigma_{ij}^{n-1}+E_{ijkl}\left(\Delta \varepsilon_{kl}-\Delta \varepsilon_{kl}^{p}-\Delta \varepsilon_{kl}^{f}\right)$$

$\Delta \varepsilon_{ij}^{p}$ is the increment of plastic strain, and $\Delta \varepsilon_{ij}^{f}$ is the increment of fracturing strain. The Rankine failure criterion for concrete cracking is applied with stresses converted in the material directions:

(3) $$F_{i}^{f}=\sigma_{ii}^{\prime t}-f_{ti}^{\prime }\leq 0$$

where $\sigma_{ii}^{\prime t}$ is the trial stress, and $f_{ti}^{\prime }$ is the trial tensile strength in the material direction *i*. The crack opening w is calculated from the total value of the calculated fracturing strain ${\hat{\varepsilon }}_{kk}^{\prime f}$ in the direction k plus the current increment of the fracturing strain Δλ, the sum being multiplied by the crack band size $L_{t}$ as in Equation (4):

(4) $$w_{k}=L_{t}\left({\hat{\varepsilon }}_{kk}^{\prime f}+\Delta \lambda \right)$$

$L_{t}$ is calculated as the size of the element projected onto the crack direction. Given the user-defined, strain-hardening material law, the crack width is adjusted according to a predefined localization strain $\varepsilon_{loc}^{t}$ and characteristic length, also described as band width limiter, $L_{ch}^{t}$, as in Equation (5):

(5) $$w_{k}=\left({\hat{\varepsilon }}_{kk}^{\prime f}-\varepsilon_{loc}^{t}\right)\frac{L_{t}}{L_{ch}^{t}}$$

The constitutive law of SHCC represents the single-crack opening behavior as derived experimentally. The location of fracture localization in the model specimen resulted from the probabilistic and axial fluctuation of the tensile strength. Given that the probabilistic parameters only varied axially along the specimen, the fracture zone was limited to one transverse row of elements, thus allowing the characteristic length to be defined equal to the element size. In this way, the pre-peak and post-peak response of the SHCC elements strictly followed the corresponding user-defined constitutive laws as presented in Section 2.2.

Given that the work at hand dealt exclusively with simulations of uniaxial tension experiments, the fixed crack model was applied with the default value of the shear retention factor [36,37,38]. Similarly, the crack closure stiffness was set to its default value, implying unloading behavior parallel to the elastic stiffness. Refining this parameter would be necessary to model the partial crack closure accurately during the formation of new cracks, softening, or due to cyclic loading [35].

### 2.2. SHCC—Constitutive Law

The reference SHCC in this study consisted of a high-strength matrix and 6 mm long ultra-high molecular weight polyethylene fibers (UHMWPE) in a volume fraction of 2% [39,40]. The composite yields an average compressive strength of 140 MPa and Young’s modulus of 29 GPa [39,41]. The average quasi-static tensile strength as derived on the specimen geometry modeled in the current work is approximately 6.7 MPa, and the average macroscopic strain at failure localization is approximately 1.3% [13,14]. Smaller SHCC specimens of identical compositions yield considerably lower crack spacing and higher strain capacity [39,40], this being traceable back to the strong influence of heterogeneity, size, and structural effects on the apparent material properties of SHCC.

**Figure 2:**
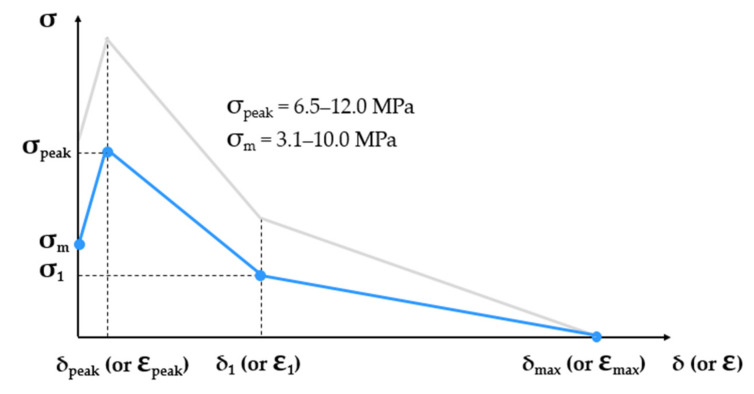
Qualitative representation of the post-elastic constitutive law of SHCC in tension including the varying matrix strength and tensile strength within predefined ranges.

The multilinear constitutive tensile law of SHCC in the post-elastic stage represents a simplified single-crack opening law and was derived by interpolating experimental data from different studies by the authors on notched and even specimens; see Figure 2 [13,14,42]. The matrix strength $\sigma_{m}$ and the tensile strength $\sigma_{peak}$ are variable material parameters. The ranges indicated in Figure 2 are partly empirical (lowest values) and partly hypothesized (highest values) due to the lack of experimental evidence regarding the strongest cross-sections in common SHCC specimens. The definition of the input laws of single SHCC elements imposed the condition of strain-hardening, i.e., tensile strength higher than the matrix strength. Given the partial overlap of the corresponding ranges of values, the violation of this condition was prevented by adjusting the axial fluctuation of the matrix strength and tensile strength along the model specimen, as explained in Section 3. In terms of stress levels, the blue curve in Figure 2 is representative of plain SHCC specimens according to the rule of the weakest link.

The constitutive strain relationships of the model are derived by dividing the presented deformation (crack opening) values to the element size of 3 mm. The crack opening at peak stress $\delta_{peak}$ has a constant value of 100 µm, and it corresponds to an equivalent strain at peak of 3.3%. The softening branch was defined based on $\sigma_{1}$ (stress at the kink point) and $\delta_{1}$ (crack width at the kink point), which are ratios of $\sigma_{fc}$ and $\sigma_{peak}$ according to Equations (6) and (7). The values of $SF_{1}$ and $SF_{2}$ are given in Table 1. The crack width at vanishing crack-bridging $\delta_{max}$ was defined as half of the fiber length, i.e., 3 mm.

(6) $$\sigma_{1}=SF_{1}\cdot \sigma_{peak}\;\mathrm{for}\;\delta =\delta_{1}$$

(7) $$\delta_{1}=SF_{2}\cdot \left(\delta_{max}-\delta_{peak}\right)$$

The strain values presented in Table 1 are only valid for single elements and not for the composite specimens, in which the selective multiple cracking led to considerably lower macroscopic deformations; see Section 3. Because the finite elements were designed to accommodate the elongation equivalent to one single crack, the term first-crack stress in this paper refers only to the model composite specimens, while the term matrix strength refers to the finite elements.

As facilitated by the axial fluctuation of the matrix strength and tensile strength, 50 monitoring points were set along the model specimen, i.e., one point for every row along the gauge portion, for deriving the crack widths under load. The corresponding crack widths were averaged over all the cracked elements and plotted next to the macroscopic stress–strain curves. In this way, the average crack width in the strain-hardening phase could be compared to that derived in the reference tension experiments.

### 2.3. Continuous Reinforcement

The reference continuous reinforcement in this study is a carbon textile produced by V. Fraas GmbH (Helmbrechts, Germany) under the brand name TUDALIT-BZT2 [43] and carrying the index T1 in this work. Its mechanical and geometric properties in the warp, i.e., principal and direction, are summarized in Table 2 based on the data by the producers and from experimental investigations by the authors [13,14,15].

In the context of structural strengthening against impact loading, the strengthening layers should ensure a strong confinement of the strengthened element [12] but at the same time exhibit a large capacity for energy dissipation in extreme loading cases. This implies that not only the SHCC but the continuous reinforcement as well should yield high inelastic deformability. As a model reinforcement of high elongation capacity, a UHMWPE textile was produced with identical geometric properties and impregnation material as the reference carbon textile [14]. The properties of the UHMWPE filaments Dyneema SK75 according to the producer (DSM, Heerlen, The Netherlands) are given in [44]. In both cases, the multi-filament yarns were impregnated with a watery styrene–butadiene based dispersion for ensuring adhesion and effective composite action among the single filaments within the yarns and with the surrounding cementitious material.

**Figure 3:**
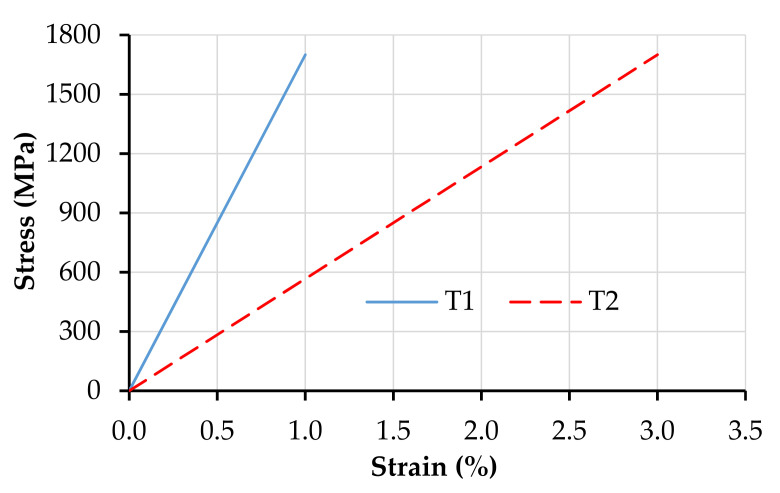
Tensile constitutive laws of the continuous reinforcement under investigation.

The continuous, unidirectional reinforcement was discretized by truss elements CCIsoTruss (type 2D) of the Reinforcement material model. These are isoparametric elements integrated by Gauss integration at one point with linear interpolation. The reinforcement truss elements have neither bending nor shear stiffness. Figure 3 presents the tensile constitutive laws of the discrete reinforcement.

Whereas the numerical model T1 represents accurately the properties of the carbon warp yarns, the geometric features and the tensile strength of T2 differ from those presented in [14]. For a systematic parameter study, in this work, they were set to be equal to the corresponding properties of T1. The interfacial equilibrium condition and stress-state in the embedded truss elements were derived based on the geometric cross-section parameters given in Table 2.

### 2.4. Bond

**Figure 4:**
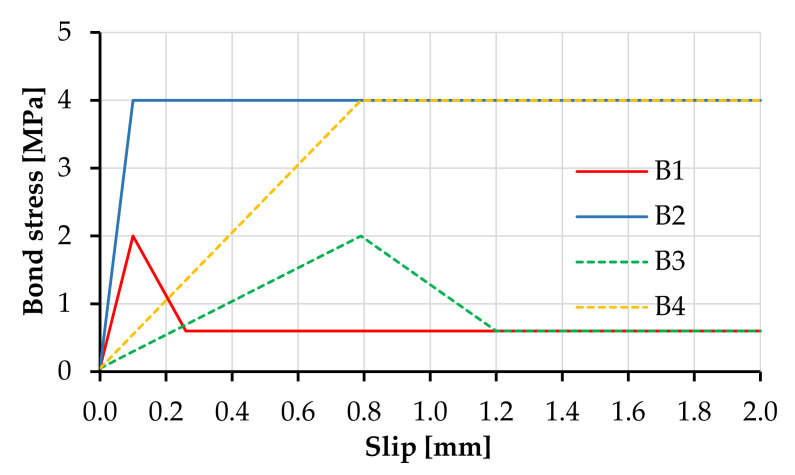
Numerical bond stress-slip laws of the continuous reinforcement.

The bond-slip was modeled with bond elements between the truss elements and the solid elements and defined by the Bond for Reinforcement material model [45]. The defined bond-slip relationships were constant parameters along the entire length of the modeled element and were defined according to the pullout experiments presented in [14,15,46]. These are presented in Figure 4 as B1 for the carbon textile and B3 for the equivalent polymer textile.

The reference bond-slip relationships assume a simplified linear relationship to the slip strength followed by a stress drop after the critical slip and subsequently by a constant stress pullout. To avoid ill-conditioning, the input bond strength vs. slip law assumed an elastic range with no slip up to bond stress of 0.05 MPa; see Figure 4.

In the previous study, the effect of bond strength was experimentally investigated by applying an additional coating of epoxy resin and sand to the yarns, which substantially increased the bond strength and altered both the tensile properties and fracture behavior of the fiber-reinforced composites [14,47]. In the case of the UHMWPE textile, the additional coating influenced the elongation behavior of the textile itself [14]. However, the high bond strength caused premature yarn rupture in pullout experiments and did not allow the derivation of the bond-slip relationships. For this reason, the alternative bond properties analyzed in the frame of the numerical parameter study involved an adhesive bond strength twice higher than that of the reference bond B1 and B3, followed by a constant stress pullout with no stress drop after the critical slip displacement; see B2 and B4 in Figure 4. Although not quantitatively comparable to the experimental data, the numerical results of B3 and B4 offered a qualitative evidence on the effect of bond strength on the tensile behavior of hybrid fiber-reinforced composites. The numerical parameters of the bond-slip relationships are summarized in Table 3.

Besides the plain SHCC model, the parameter combinations, i.e., numerical material compositions, investigated in this work were: (1) SHCC-T1-B1, equivalent to M-PE-T in [13]; (2) SHCC-T1-B2, equivalent to M1-PE-TE in [47]; (3) SHCC-T2-B3; and (4) SHCC-T2-B4. The parameter combinations (3) and (4) are qualitatively equivalent to composites reinforced by a polymer textile, but they do not yield a quantitative comparison.

## 3. Probabilistic Material Parameters

**Figure 5:**
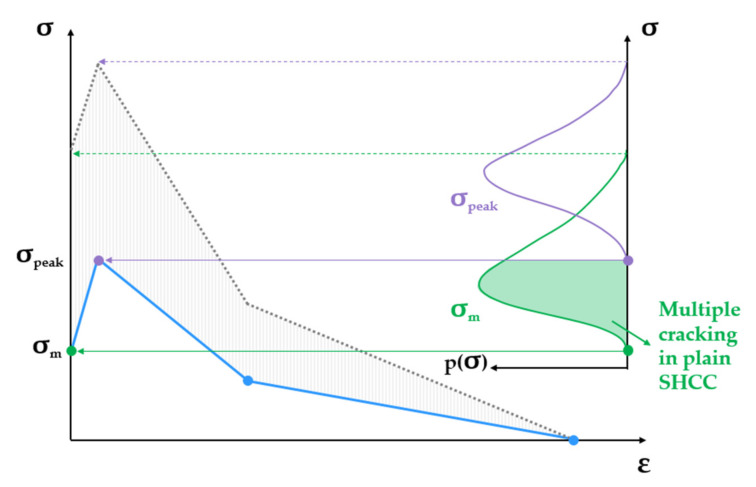
Schematic representation of the probability distribution of the matrix strength and tensile strength of SHCC and the minimum parameter values defining the tensile behavior of plain SHCC according to the blue multilinear law. The representation refers to the post-elastic regime.

The inherent material variability of SHCC in terms of flaw size and fiber distribution determines their effective tensile strength and ductility [48]. The material inhomogeneity in real-scale applications is expected to be considerably more pronounced, and its negative effects can be partly mitigated in combination with continuous reinforcement. Proper material design in this respect should allow exploiting the potential ductility of SHCC and avoid premature crack localization [12]. In numerical simulations, this effect can be highlighted by involving a probabilistic representation of the matrix strength and tensile strength of SHCC, as presented in Figure 5.

The strength parameters of plain SHCC as shown by the blue curve in Figure 5 are determined by the lowest values of the matrix strength and tensile strength and by the shape of the distribution functions of these parameters. Given predefined minimum values, distribution functions of the matrix strength with a lower median should result in a higher extent of multiple cracking than in the case of a larger median. This effect is presented in Figure 5 by the green hatched area, which increases if the distribution function of the matrix strength yields a lower median.

It should be mentioned that the theoretical minimum crack spacing in plain SHCC is determined by the short fibers’ transmission length, which represents the tensile stress increment in the matrix with increasing distance from the crack flank [49]. However, the analytically derived transmission length is an idealized parameter and hardly achievable in common SHCC samples.

The Weibull function [50] was selected to define the probability distribution of the varying material parameters since its shape can be adjusted to match different distributions and demonstrate the above-formulated statements within a parameter study. Additionally, the Weibull distribution is commonly adopted to represent the mechanical strength of materials [31,51,52]. The three-parameter Weibull distribution was analyzed in this work, with the corresponding probability density function $f\left(x\right)$ presented in Equation (8) and cumulative distribution function $F\left(x\right)$ in Equation (9). The following conditions apply: $f\left(x\right)\geq 0,\;x\geq 0,\;x\geq \gamma ,\;\beta >0,\;\alpha >0,\;\mathrm{and}\;\gamma =\mathbb{R}.$

(8) $$f\left(x\right)=\frac{\beta }{\alpha }{\left(\frac{x-\gamma }{\alpha }\right)}^{\beta -1}e^{\left[-{\left(\frac{x-\gamma }{\alpha }\right)}^{\beta }\right]}$$

(9) $$F\left(x\right)=1-\exp[-(\frac{x-\gamma }{\alpha }{)}^{\beta }]$$

The value of the shape parameter β, also the slope parameter, controls the frequency distribution of the randomized parameter x. The value of the scale parameter α, characteristic life, is responsible for the width of the distribution range, given a constant β. The location parameter γ locates the lower bound of the predefined range of x.

The probabilistic material properties were generated in a preprocessing module. Firstly, the pseudo-random numbers $R_{u}$ that follow a uniform distribution with values from 0 to 1 were generated. Subsequently, the cumulative probability function of the Weibull distribution $f\left(x\right)$ was equated to $R_{u}$ according to [53], resulting in the array of numbers $R_{w}$ as shown in Equation (10):

(10) $$R_{w}=\alpha \left[-ln{\left(1-R_{u}\right)}^{\frac{1}{\beta }}\right]+\gamma$$

The parameters to be defined besides the Weibull parameters are the size of the array with the minimum and the maximum values. In the presented study, the material inhomogeneity was only defined in the longitudinal direction of the model specimens, i.e., the properties did not vary transversally. This was intended to simulate the steady-state cracking in SHCC elements with small cross-sections, which implies instant crack propagation through the entire cross-section shortly after crack initiation. Given that the effective size of the random arrays generated in this work was equal to the number of elements along the gauge portion of the model specimen, i.e., 50, the probability of the smallest generated number to be equal to the predefined inferior boundary was extremely low, especially with larger *β*. For this reason, the location parameter was adjusted to shift the generated arrays to the predefined inferior boundaries.

**Figure 6:**
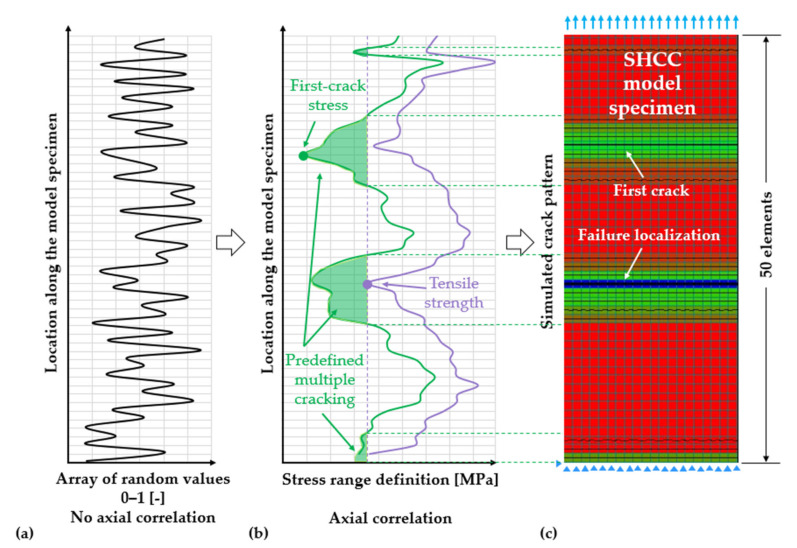
(**a**) Elementwise distribution of axially non-correlated probabilistic values generated within normalized boundaries. (**b**) Axial correlation and range definition for the matrix strength and tensile strength. (**c**) Effect of the probabilistic distribution and axial fluctuation of matrix strength and tensile strength, as well as of the range overlap of these parameters on the degree and regions of multiple cracking in a plain SHCC model specimen.

The initially generated randomized values and their axially non-correlated fluctuation are schematically presented in Figure 6a. These sets of numbers were transposed into predefined stress ranges depending on the material parameter to be represented, as shown in Figure 6b. The next step consisted in the cross-correlation [54] of the matrix-strength and tensile strength as well as in their axial correlation, as shown in Figure 6b. The cross-correlation did not imply an explicit interdependence of the matrix strength and tensile strength but mainly targeted a congruent pattern of the green and purple fluctuations in Figure 6b. In this way, strain-hardening was ensured in all finite elements along the model specimen.

The probabilistic parameters were axially correlated according to a predefined correlation length, which is a range that the algorithm uses to group the adjacent values spatially for steady variation. From the example in Figure 6b, the correlation length is approximately equal to 45 mm or 15 elements. There is no experimental evidence on the spatial variability of the matrix strength and tensile strength in the reference SHCC samples. However, given that only axial variability was considered in this work, the representation in Figure 6b is presumably more realistic than that in Figure 6a, since the axial fluctuation must represent an average of the variability in the other directions, which levels out over the width and thickness of the specimens.

The probabilistic material parameters were assembled into readable input files for the FE software, which included the user-defined constitutive relationships attributed to specific elements according to their location in the model SHCC specimen. Figure 6c shows the effective number of cracked elements at failure localization corresponding to exemplary probability distribution and axial fluctuation of matrix strength and tensile strength in Figure 6b.

Note that the axial fluctuation of the probabilistic parameters is irrelevant to the effective extent of multiple cracking in plain SHCC. With no cross-correlation, the predefined regions of multiple cracking, the green hatched areas in Figure 6b, would be distributed along the specimen as in Figure 6a. However, their total area would not change, since that only depends on the number of elements having matrix strength lower than the tensile strength of the weakest element in the specimen.

Since the experimental data only provide evidence on the far-left tails of the distribution functions, the probability distributions presented in this section are not entirely empirical and are only intended to highlight the importance of probabilistic numerical modeling of such composites.

## 4. Results and Discussion

### 4.1. Influence of the Probability Distribution Functions on the Macroscopic Composite Response

**Figure 7:**
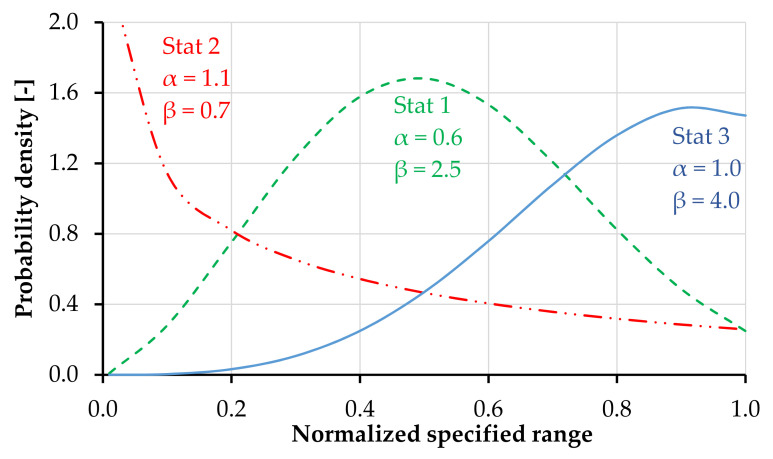
Different Weibull probability density functions analyzed within the parameter study.

Figure 7 presents the probability density functions analyzed, including the corresponding scale and shape parameters. These were defined to mirror three different cases with distinguishable effects on the tensile response of the model specimens. The predefined stress range of the matrix strength was 4–10 MPa and that of the tensile strength was 6.5–12 MPa. By adjusting the location parameter *γ*, the generated values fit accurately the predefined lower boundaries.

**Figure 8:**
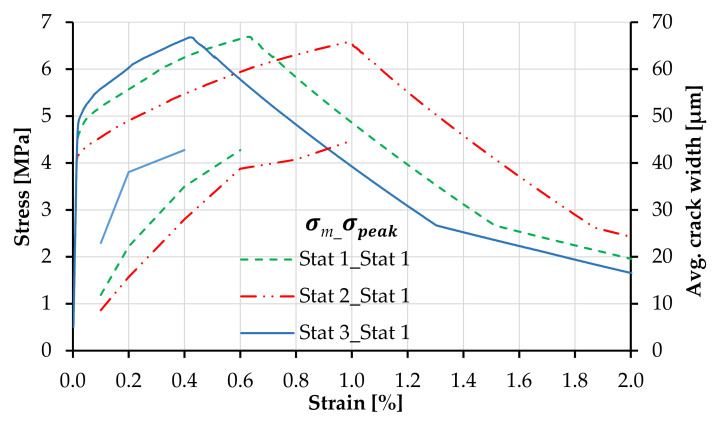
Tensile stress–strain curves corresponding to plain SHCC with different distribution functions of matrix strength and constant distribution function of tensile strength.

Figure 8 presents the effect of the statistical distribution of matrix strength on the shape of the tensile stress–strain curves of plain SHCC, including the strain capacity and crack width. The legend first indicates the probability distribution of the matrix strength and secondly that of the tensile strength. The curves follow the same color and line codes as of the probability distributions in Figure 7. The same correlation length and axial distribution were applied to all models.

**Figure 9:**
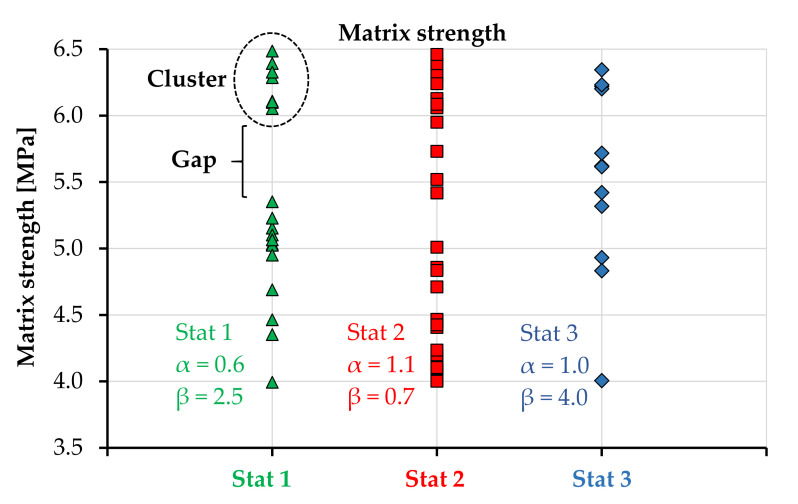
Number and spacing of the matrix strength values in the generated arrays up to the macroscopic tensile strength (6.5 MPa) depending on probability distribution.

The distribution function of matrix strength influences not only the shape of the strain-hardening branch but also strain capacity and average crack width. If the distribution with a low median is applied to the matrix strength such as Stat 2, the SHCC model yields a higher number of cracks and consequently a lower average crack width in the strain-hardening phase. Contrarily, if the distribution function of the matrix strength yields a larger median, such as Stat 3, fewer elements are loaded beyond the elastic range, and the resulting average crack width is higher in the initial post-crack stage. The median and average values of the generated arrays defining the matrix strength and tensile strength depending on the probability distribution function are summarized in Table 4. The effective number of generated values for matrix strength depending on the Weibull function is presented in Figure 9 up to the macroscopic composite strength of 6.5 MPa.

Given the different number of cracks forming at different stress levels, as shown in Figure 9, the average crack widths differ among the three SHCC models at the beginning of the strain-hardening phases, while at failure localization (6.5 MPa), the average crack widths are approximately 45 µm in all three parameter combinations; see also Table 5.

Although the lower boundaries of the distribution functions are identical for all SHCC models in Figure 8, it might appear that the first-crack stress of the parameter combination Stat 3_Stat 1 is higher when compared to the other parameter combinations. This results, however, only from the shape of the matrix strength distribution function, which yields fewer values along the far-left tail, as in Figure 9, and an earlier imposed yielding of the stronger elements, causing a steeper ascending branch.

**Figure 10:**
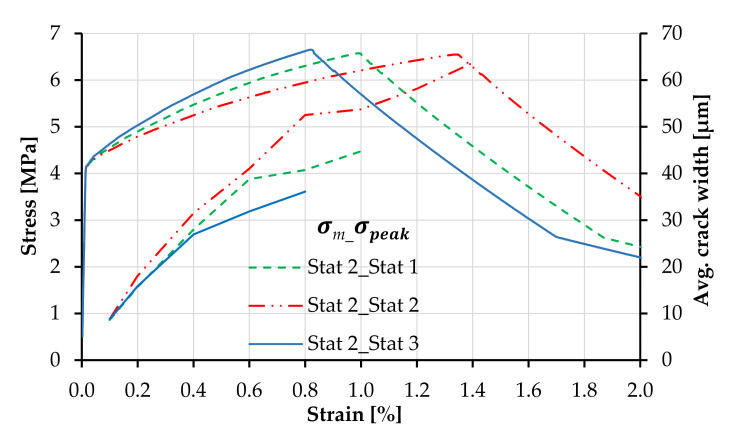
Tensile stress–strain curves corresponding to plain SHCC with different distribution functions of tensile strength and constant distribution function of matrix strength.

The unsteady crack width curves in Figure 8 and Figure 10 result from the uneven spacing between the individual values in the generated arrays, as demonstrated in Figure 9. The cracks form in unevenly distributed clusters with small stress increments within the clusters and larger stress increments between clusters. In this way, the formation of a new series of cracks causes specimen relaxation and stabilization of the average crack width under increasing macroscopic strain.

Figure 10 presents the macroscopic stress–strain relationships of plain SHCC models with different distribution functions of tensile strength. In this example, the distribution function of the matrix strength was maintained constant as Stat 2. Despite the predefined number of cracks prior to softening, the probability distribution of the tensile strength has a strong effect on the macroscopic strain at localization. This effect can be traced back to the median strain-hardening modulus across the elements in the SHCC model specimen, resulting in different average crack widths, as shown in Table 6.

**Figure 11:**
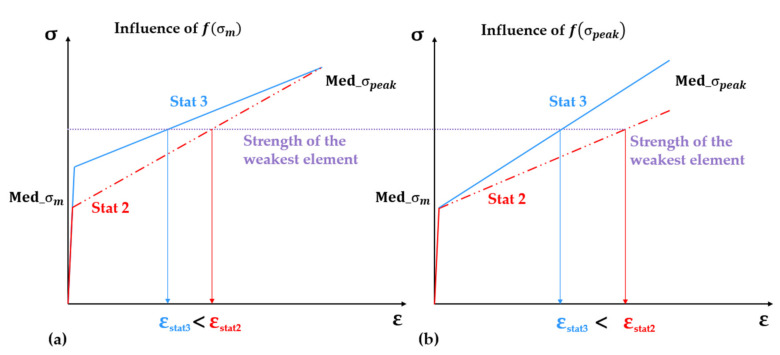
Effect of the shape of the probability distribution of (**a**) matrix strength and (**b**) tensile strength on the median values over all the finite-elements along the SHCC model specimens and, as a result, on the macroscopic strain at failure localization.

As graphically illustrated in Figure 11a, given a certain minimum strength value, which defines the global strength of the composite, i.e., the strength of the weakest element, Stat 2 yields a lower median matrix strength compared to Stat 3, also from Table 4, which results in higher strain values upon reaching the macroscopic tensile strength. This effect is magnified by the varying extent of multiple cracking, as shown in Table 5. On the other hand, if the distribution function of the tensile strength yields a lower median (Stat 2), the median strain-hardening modulus of the model SHCC specimen is lower, resulting in the higher strain at a failure localization, as shown in Figure 11b.

### 4.2. Reference SHCC Model

**Figure 12:**
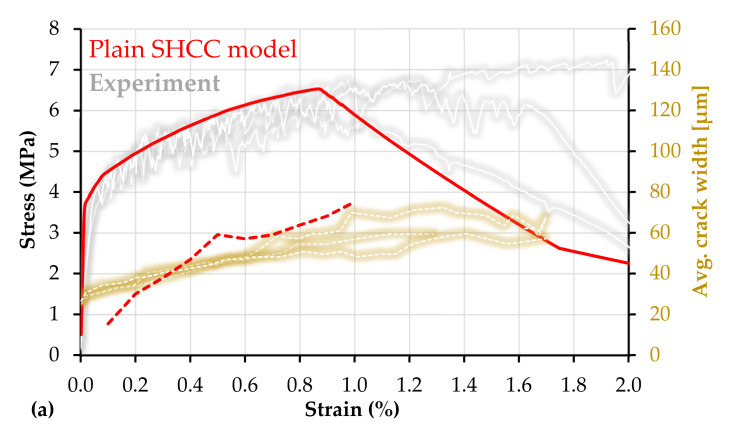

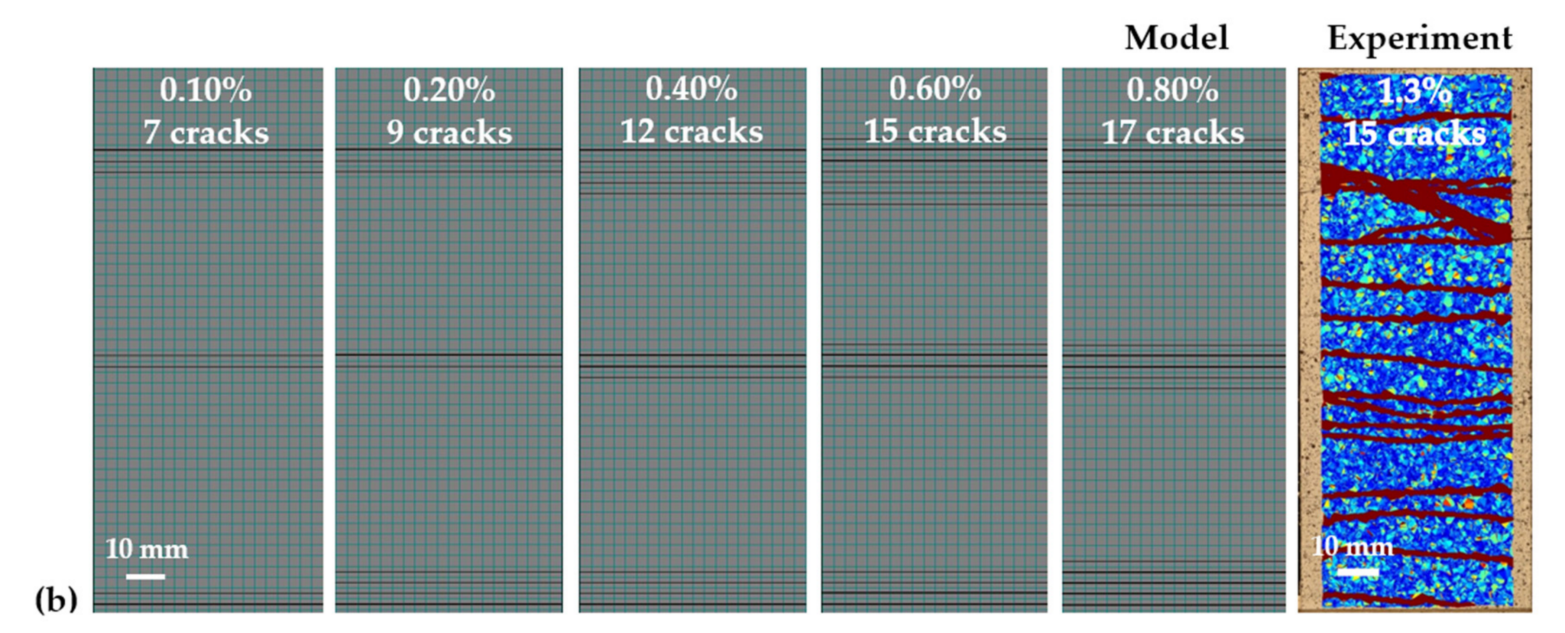
(**a**) Macroscopic stress–strain curve (continuous) and average crack widths (dashed) of the reference SHCC model composite with experimental results adopted from [13]; (**b**) crack pattern and sequence in the loaded SHCC model and a representative experimental crack pattern based on DIC analysis.

Within the parameter study presented in Section 4.1, the strength and probabilistic parameters of a reference SHCC model were defined, parameters that approximate the average response of the plain SHCC specimens in [13,14]. Plain SHCC exhibit limited robustness in terms of strain capacity, and the experimental results in [13,14] differ slightly, despite involving the same material composition, production, and testing techniques. For this reason, the results in [13] were adopted as the exemplary experimental basis in Figure 12a. The experimental average tensile strength was 6.8 MPa, and the average strain capacity was 1.3%. The average crack width at peak load was approximately 60 µm, while the average number of cracks in the gauge portion at failure localization was 15. These were the reference experimental properties taken into account while adjusting the strength and probabilistic parameters of the model, which are summarized in Table 7.

The stress–strain curve of the reference SHCC model is presented in red in Figure 12a next to the corresponding experimental curves in gray. Additionally, the average crack width of the model with increasing deformation is presented comparatively next to the average crack widths from the corresponding experiments in [13]. The corresponding crack pattern in the model specimen is presented in Figure 12b, which also shows the crack pattern in a representative SHCC specimen at failure localization. The experimental crack pattern and width were assessed by means of Digital Image Correlation (DIC) as described in [14].

Note that the reference model parameters were not intended to match the experimental curves perfectly, but rather to offer a numerical basis for assessing the influence of continuous reinforcement in comparison to the equivalent experimental results. As shown in Figure 12a, the model underestimates the average strain capacity of the experimental specimens and yields a certain discrepancy in respect of average crack width. Thus, further refinement of the probabilistic parameters should be carried out in future studies.

**Figure 13:**
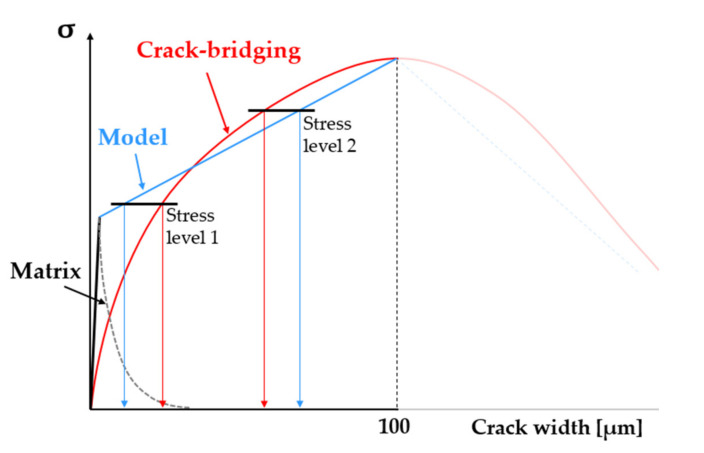
Difference between the numerical constitutive law (blue) and real crack-bridging relationship (red) causing a discrepancy between the average crack widths of the model and experimental SHCC specimens.

The same applies to the generated arrays, which should yield a more even distribution of the single values within the predefined ranges as compared to the arrays presented in Figure 9. Finally, besides the crack closure stiffness of SHCC, i.e., unloading behavior, as described in Section 2.2, the simplified constitutive law of SHCC might also cause the discrepancy of average crack widths. From a structural point of view, the crack-bridging stiffness of SHCC is lower than the stiffness of the matrix, which results in stress drops upon crack formation under deformation controlled loading. This structural relaxation was not implemented into the numerical constitutive law, which resulted in an underestimation of the crack width at lower stress levels, as shown schematically in Figure 13. At stress level 1, the model elongation is lower than the equivalent crack opening. The opposite effect can be encountered at higher stress levels, due to the non-linearity of the crack-bridging relationship. A possible refinement in this respect could be carried out as in [29].

### 4.3. Hybrid Fiber Reinforced Composites—SHCC-T1-B1

**Figure 14:**
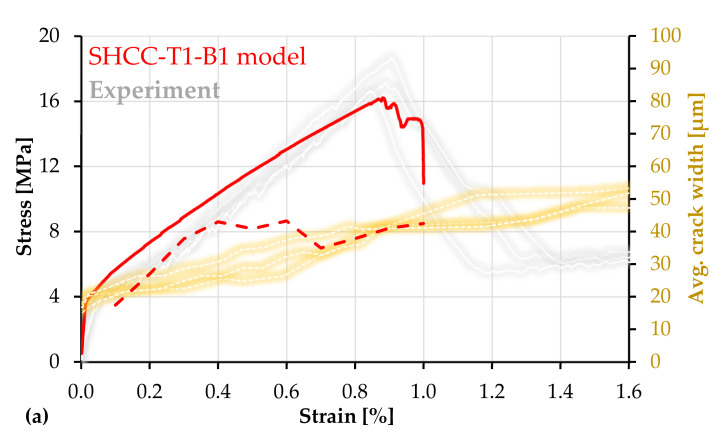

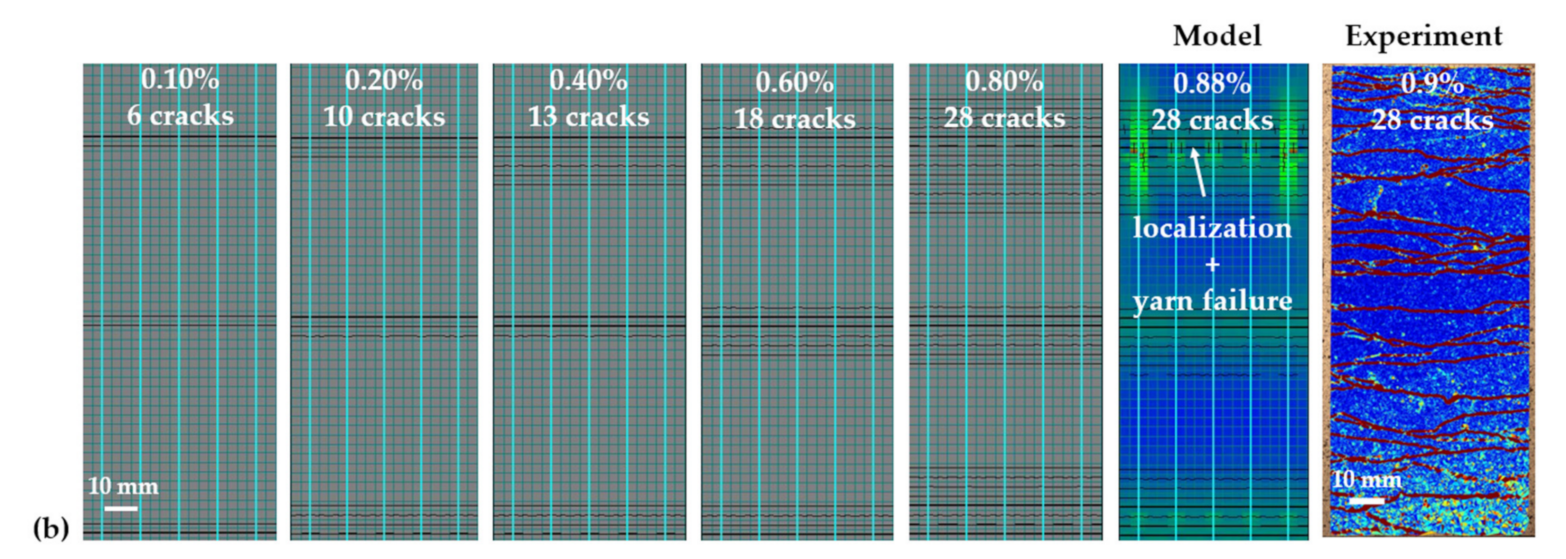
(**a**) Macroscopic stress–strain curve (continuous) and average crack widths (dashed) of SHCC-T1-B1 model composite with experimental results adopted from [13]; (**b**) crack pattern and sequence in the loaded model including the principal stresses in SHCC at yarn failure and a representative experimental crack pattern based on DIC analysis.

The experimental combination M-PE-T from [13] represents a high-strength SHCC with an as-received carbon textile, i.e., with no additional coating, and it is the reference for the numerical model SHCC-T1-B1 in this work. The relatively weak yarn–SHCC bond in this material combination allowed yarn rupture without crack localization in SHCC. As shown by the gray curves in Figure 14a, the yarns failed on average at a composite stress of 17.5 MPa, which caused a pronounced load drop, but did not impede further deformation and multiple cracking in the SHCC. The yarns failed consecutively in different locations along the specimens and the telescopic yarn elongation after failure prevented pronounced localized deformations in the surrounding SHCC. More than that, despite having failed, the embedded yarns stabilized the specimens against in-plane rotation and facilitated uniform crack opening and a strain at failure localization in SHCC of more than 3%, which was substantially higher than that yielded by the plain SHCC specimens. However, the numerical simulations do not reproduce these phenomena and the model–experiment comparison is only possible up to yarn failure.

In the experiments, the average load-bearing capacity of M-PE-T was 19.6 kN, while the average load-bearing capacity of the bare textile was 14.7 kN. The difference of 4.9 kN can be partly attributed to the residual yarn anchorage at failure but mainly to the crack-bridging of the short fibers in SHCC, which did not demonstrate localization on yarn failure. Although to a lower extent, this effect was also reflected by the numerical simulations. The load-bearing capacity of the continuous reinforcement in the model is 15.3 kN (equivalent to a composite stress of 12.7 MPa), while the composite load-bearing capacity as shown in Figure 14a is 19.2 kN, composite stress of 16.2 MPa. The difference of 4.1 kN (3.5 MPa) is the additional contribution of SHCC. This was confirmed by the detailed crack analysis in the region of failure localization.

In the simulation presented in Figure 14a, at peak load (0.9% strain), SHCC reached a maximum crack width of 100 µm in the region of failure localization. Upon yarn failure at 1.0% strain, the maximum crack width in SHCC was 158 µm, in the softening stage, and the stress distribution in the respective cross-section was not uniform, as shown in the highlighted model image in Figure 14b. The color code indicates the principal stresses in SHCC: red = 1.4 MPa up to blue = 7.9 MPa. Upon yarn failure, the composite stress level was 14.9 MPa, which is 2.2 MPa higher than the load-bearing capacity of the continuous reinforcement. Thus, the additional contribution of 2.2 MPa (2.6 kN) corresponds to the average tensile stress in SHCC along the localization crack.

The experimental plain SHCC specimens presented in Figure 12a reached a crack spacing at peak load of 11.5 mm on average, while in combination with carbon textile, the crack spacing at yarn failure was in the range of 5.2 mm, which indicates approximately 28 cracks within the gauge portion. Thus, as shown in Figure 14b (model and experiment), the simulation yielded an accurate estimation of multiple cracking. Additionally, the simulation captured the decrease in average crack width of 75 µm at failure localization for the plain SHCC model to 42 µm for SHCC-T1-B1, which is also in agreement with the experimental results.

As mentioned in Section 4.1, the unsteadiness of the average crack width curves is related to the modeled cracking sequence, which needs further refinement. Figure 14b shows that the hybrid fiber-reinforced model yielded 18 cracks at a macroscopic strain of 0.6%, at which the crack width discrepancy compared to the experiments is the highest. At 0.7% strain, a new cluster of cracks formed, allowing the average crack width of the model to approach the experimental one. Another discrepancy between the model and experiments is related to the shape and slope of the stress–strain curves in the strain-hardening phase. It may be assumed that a more accurate adjustment of the probabilistic parameters would achieve superior accuracy both in terms of macroscopic stress–strain relationships and in terms of average crack width.

### 4.4. Hybrid Fiber Reinforced Composites—SHCC-T1-B2 (Influence of Bond Strength)

The influence of the bond strength between SHCC and the carbon textile was investigated within the experimental parameter study presented in [47]. The extra coating applied to the carbon textile consisted of epoxy resin and sand. It increased the bond strength substantially and completely altered the tensile properties and fracture behavior of the corresponding composite specimens M-PE-TE [47]. M-PE-TE yielded a tensile strength of 26.7 MPa, 50% higher than that of M-PE-T, and a strain capacity of 1.4%, 55% higher in comparison to the strain at peak load of M-PE-T. The average crack spacing in M-PE-TE was 3.9 mm, meaning approximately 38 cracks within the gauge length, while the average crack width at peak load was on the order of 45 µm. Thus, the strong bond ensured superior synergetic action between SHCC and textile when compared to M-PE-T, leading to higher tensile strength, pre-peak deformability, and crack control.

**Figure 15:**
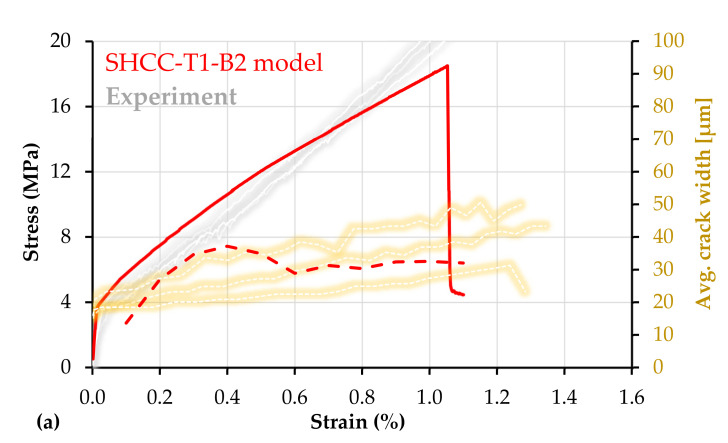

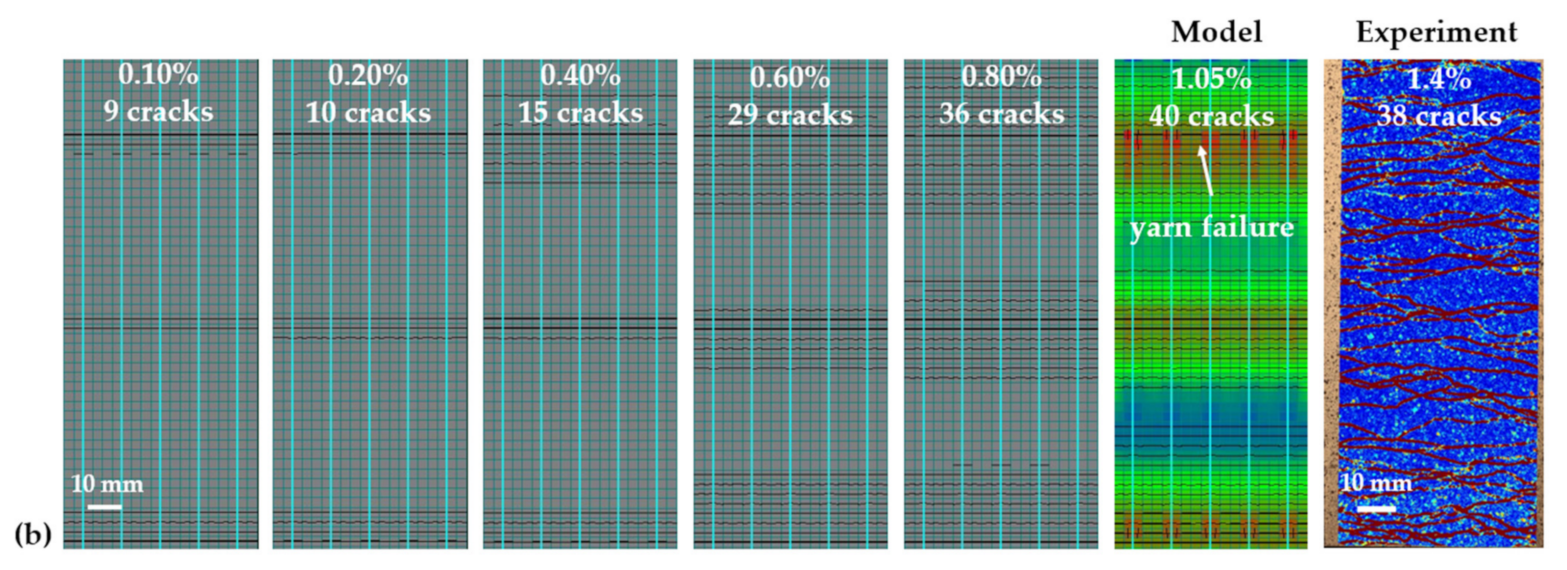
(**a**) Macroscopic stress–strain curve (continuous) and average crack widths (dashed) of the SHCC-T1-B2 model composite with experimental results adopted from [47]; (**b**) crack pattern and sequence in the loaded model including the principal stresses in SHCC at yarn failure and a representative experimental crack pattern based on DIC analysis.

Given the high tensile strength, even in comparison to M-PE-T, it can be stated that the strong bond facilitated a superior contribution of the short fibers to the peak load-bearing capacity of M-PE-TE. This effect was partially mirrored by the corresponding numerical model in Figure 15a. The tensile strength of SHCC-T1-B2 (18.5 MPa) is higher than that of SHCC-T1-B1 (16.2 MPa). The less pronounced enhancement in strength compared to the experimental results is related to the input bond strength, which was considerably lower than that of the extra-coated carbon yarns in the experiments.

Besides the increase in composite strength, the simulations reproduced accurately the pronounced increase in multiple cracking, shown in Figure 15b, compared to the plain SHCC model and to SHCC-T1-B1. The number of cracks yielded by the model at peak load is similar to the average number of cracks of the experimental specimens.

As opposed to M-PE-T, the strong bond in M-PE-TE did not allow delamination or slip of the rupturing yarns, causing immediate localization in SHCC. This effect was implicitly reflected in the numerical simulations. As opposed to SHCC-T1-B1, SHCC-T1-B2 yielded no localization in SHCC up to peak load. The maximum crack width at peak load was 98 µm, and the corresponding crack was located in the middle of the composite specimen away from the location of yarn failure.

Whereas failure in SHCC-T1-B1 occurred at 1.0% macroscopic strain, as taken from Figure 14a, in the case of SHCC-T1-B2 the strain at failure localization exceeded 1.0%; see Figure 15a, this being in agreement with the experimental results. The fact that the macroscopic strain capacity of the composite gauge portion can be higher than the nominal strain capacity of the longitudinal reinforcement is related to the assessment principle of the macroscopic strain both in experiments and simulations. As described in Section 2.1, the macroscopic strain was calculated according to the relative displacements of the solid element nodes at the margins of the gauge portion; see Figure 1. Given that the no-slip condition between the truss elements and solid elements was defined at the boundary nodes of the entire model specimen, the elongation of the gauge portion includes the elongation and slip of the truss elements inside the anchorage portions, outside of the gauge portion, over the entire length of 160 mm. A similar effect was also observed in [14,47]. Disabling the slip of the truss elements at the ends of the gauge portion would result in lower macroscopic strains at localization, but this would not correspond to the experimental setup and would not allow a direct comparison.

### 4.5. Hybrid Fiber Reinforced Composites—Influence of Yarn Stiffness and Elongation Capacity

**Figure 16:**
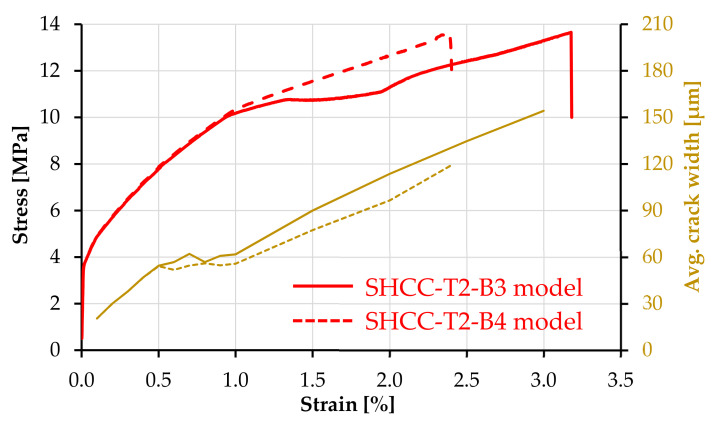
Macroscopic stress–strain curves and average crack widths of the SHCC-T2-B3 (continuous) and SHCC-T2-B4 (dashed) model composites.

The stress–strain curves and average crack widths of SHCC-T2-B3 (ordinary bond) and SHCC-T2-B4 (strong bond) are presented in Figure 16. The main difference regarding the tensile behavior of these two model composites is related to the considerably lower strain at failure localization of SHCC-T2-B4 as a result of lower average crack width. The average crack width of the two composites started diverging at 0.5% macroscopic strain, whereas, at 1.0% strain, the crack localization caused a more pronounced increase in average crack width with increasing deformation. The maximum number of cracks in SHCC-T1-B3 was 22, while in SHCC-T1-B4 it was 25. This extent of multiple cracking was reached up to localization. Thus, despite the high elongation capacity of the continuous reinforcement, the truss elements did not trigger the formation of more cracks beyond the localization strain of plain SHCC. Furthermore, as opposed to the composites reinforced with T1, the increase in bond strength in the case of T2 had only a minor effect on the crack control. In fact, the limited effect of an increased bond strength was also reported in the study with UHMWPE textile [14]. However, in the named study, the composites with polymer textile did yield a considerably higher elongation capacity and multiple cracking compared to those reinforced with carbon textile, and the specimens did not show premature failure localization.

Given the early crack localization in SHCC compared to the elongation capacity of the continuous reinforcement, the tensile strength of SHCC-T2-B3 and SHCC-T2-B4 was only 13.6 MPa, which corresponds to a load-bearing capacity of 16.3 kN. The load-bearing capacity of the continuous reinforcement is 15.3 kN, meaning that the residual contribution of SHCC at yarn failure was below 1 MPa, which corresponds to a crack opening higher than 1.5 mm.

The reason for such a qualitative discrepancy when comparing the model and experimental findings is most probably related to the lower tensile strength and Young’s modulus of T2 in this work compared to UHMWPE in [14]; see Table 2. Nevertheless, the model indicates that the involvement of a softer textile with higher elongation capacity in hybrid fiber-reinforced composites is not straightforward, and further adjustments of the constituents and of their interaction are necessary to exploit the mutual deformational compatibility of the reinforcing yarns and SHCC. In this respect, additional improvements in the parameters to be investigated could include higher bond strength, smaller yarn spacing, and non-linearity of the tensile response typical for polymer fibers.

## 5. Conclusions and Outlook

The study presents a finite-element model of strain-hardening cement-based composites (SHCC) with discretized continuous reinforcement. Uniaxial tension experiments were simulated, with boundary conditions and material properties adopted from reference experimental studies on hybrid fiber-reinforced composites [13,14,15,47] in which the continuous reinforcement consisted of 2D textiles made of carbon and ultra-high molecular weight polyethylene (UHMWPE).

The SHCC material model was based on the smeared crack formulation and the constitutive law represented the single crack-opening behavior derived from experimental results. For an accurate assessment of the effect of continuous reinforcement and bond strength on the extent of multiple cracking and crack width in the hybrid fiber-reinforced composites, the matrix strength and tensile strength of SHCC were defined within a probabilistic framework following various Weibull probability distributions. This allowed for a selective multiple cracking in the model composites based on predefined distribution functions and axial fluctuation along the model specimens. The effect of the Weibull parameters on the composite response was investigated within a parameter, which demonstrated the strong influence of the probabilistic parameter definition and the importance of such an approach for achieving an accurate prediction of the composite response both with and without continuous reinforcement.

The simulations with continuous reinforcement could reliably reproduce the reference experimental results in terms of macroscopic stress–strain relationships, the extent of multiple cracking, and crack width, demonstrating that the model is suitable for extensive parameter studies on material composition and constituents’ properties.

Given the discrete representation of the continuous reinforcement, the influence of various mechanical (strain rate) and environmental (temperature) effects on the composite properties could be investigated by adjusting the properties of SHCC, continuous reinforcement, and bond based on existing experimental results. Furthermore, the model can be adapted and upscaled to simulate the strengthening performance of such composites on structural elements or to predict the mechanical performance of such composites with other types of continuous reinforcement, e.g., steel rebars.

## Figures and Tables

**Table 1:** Material parameters defining the constitutive law of SHCC.

$\delta_{peak}$	(µm)	100
$\delta_{1}$	(µm)	1160
$SF_{1}$	(-)	0.40
$SF_{2}$	(-)	0.40
$\delta_{max}$	(µm)	3000
$\varepsilon_{peak}$	(%)	3.3
$\varepsilon_{1}$	(%)	38.7
$\varepsilon_{max}$	(%)	100
$\varepsilon_{loc}^{f}$	(%)	3.3
$L_{ch}^{f}$	(mm)	3.0
$\sigma_{fc}$	(MPa)	3.7–10.0
$\sigma_{peak}$	(MPa)	6.5–12.0
Compressive strength	(MPa)	140
Elastic modulus	(GPa)	29
Poisson’s ratio	(-)	0.2

**Table 2:** Mechanical and geometric properties of the continuous reinforcement adopted in the numerical simulations.

		T1	T2
Tensile strength	(MPa)	1700	
Young’s modulus	(GPa)	170.0	56.6
Elongation at break	(%)	1.0	3.0
Effective cross-section	(mm^2^)	1.8	
Cross-sectional perimeter	(mm)	7.5	
Yarn spacing	(mm)	12	

**Table 3:** Numerical parameters defining the bond-slip relationships.

		B1 (for T1)	B2 (for T1)	B3 (for T2)	B4 (for T2)
Adhesive bond strength	(MPa)	2.0	4.0	2.0	4.0
Slip at peak load	(mm)	0.1	0.1	0.79	0.79
Slip at complete delamination	(mm)	0.26	-	1.2	-
Frictional bond strength	(MPa)	0.6	4.0	0.6	4.0

**Table 4:** Median and average values of matrix strength and tensile strength over the elements in an SHCC model specimen depending on the Weibull distribution function. These values are only representative for the arrays of the numbers analyzed in this study.

Probability Function	$\mathrm{Med}\_\sigma_{m}$ (MPa)	$\mathrm{Avg}\_\sigma_{m}$ (MPa)	$\mathrm{Med}\_\sigma_{peak}$ (MPa)	$\mathrm{Avg}\_\sigma_{peak}$ (MPa)
Stat 1	7.1	6.9	9.0	9.0
Stat 2	6.1	6.2	8.0	8.4
Stat 3	7.6	7.4	10.0	10.0

**Table 5:** Macroscopic strain at localization, average crack width, and degree of multiple cracking depending on the distribution function of the matrix strength. Data correspond to Figure 8.

Probability Functions of $\sigma_{m}\_\sigma_{peak}$	Strain at Localization (%)	Nr. of Cracks at Localization (-)	Average Crack Width at Localization (µm)
Stat 1_Stat 1	0.64	20	45
Stat 2_Stat 1	1.00	31	45
Stat 3_Stat 1	0.44	12	45

**Table 6:** Macroscopic strain at localization, average crack width, and degree of multiple cracking depending on the distribution function of the tensile strength. Data correspond to Figure 10.

Probability Functions of $\sigma_{m}\_\sigma_{peak}$	Strain at Localization %)	Nr. of Cracks at Localization (-)	Average Crack Width at Localization (µm)
Stat 2_Stat 1	1.00	32	45
Stat 2_Stat 2	1.35	32	62
Stat 2_Stat 3	0.83	32	36

**Table 7:** Strength and Weibull parameters of the reference, plain SHCC model as presented in Figure 12.

Material Parameter	Range (MPa)	Weibull Parameters		
		*α*	*β*	*γ*
$\sigma_{m}$	3.7–6.0	1.1	0.7	0.09
$\sigma_{peak}$	6.5–12	0.6	2.5	0.15

## Data Availability

The data presented in this study are available on request from the corresponding author. The data are not publicly available due to ongoing investigations.
